# Salinity stress induces the production of 2-(2-phenylethyl)chromones and regulates novel classes of responsive genes involved in signal transduction in *Aquilaria sinensis* calli

**DOI:** 10.1186/s12870-016-0803-7

**Published:** 2016-05-26

**Authors:** Xiaohui Wang, Bowen Gao, Xiao Liu, Xianjuan Dong, Zhongxiu Zhang, Huiyan Fan, Le Zhang, Juan Wang, Shepo Shi, Pengfei Tu

**Affiliations:** Modern Research Center for Traditional Chinese Medicine, Beijing University of Chinese Medicine, Beijing, 100029 China; Baotou Medical College, Baotou, 014060 China; College of Pharmacy, Zhejiang Chinese Medical University, Hangzhou, 310053 China

**Keywords:** *Aquilaria sinensis*, 2-(2-phenylethyl)chromones, Salinity stress, Transcriptome Differentially expressed gene, Signal transduction

## Abstract

**Background:**

Agarwood, is a resinous portion derived from *Aquilaria sinensis*, has been widely used in traditional medicine and incense. 2-(2-phenylethyl)chromones are principal components responsible for the quality of agarwood. However, the molecular basis of 2-(2-phenylethyl)chromones biosynthesis and regulation remains almost unknown. Our research indicated that salt stress induced production of several of 2-(2-phenylethyl)chromones in *A. sinensis* calli. Transcriptome analysis of *A. sinensis* calli treated with NaCl is required to further facilitate the multiple signal pathways in response to salt stress and to understand the mechanism of 2-(2-phenylethyl)chromones biosynthesis.

**Results:**

Forty one 2-(2-phenylethyl)chromones were identified from NaCl-treated *A. sinensis* calli. 93 041 unigenes with an average length of 1562 nt were generated from the control and salt-treated calli by Illmunina sequencing after assembly, and the unigenes were annotated by comparing with the public databases including NR, Swiss-Prot, KEGG, COG, and GO database. In total, 18 069 differentially expressed transcripts were identified by the transcriptome comparisons on the control calli and calli induced by 24 h or 120 h salinity stress. Numerous genes involved in signal transduction pathways including the genes responsible for hormone signal transduction, receptor-like kinases, MAPK cascades, Ca^2+^ signal transduction, and transcription factors showed clear differences between the control calli and NaCl-treated calli. Furthermore, our data suggested that the genes annotated as chalcone synthases and *O*-methyltransferases may contribute to the biosynthesis of 2-(2-phenylethyl)chromones.

**Conclusions:**

Salinity stress could induce the production of 41 2-(2-phenylethyl)chromones in *A. sinensis* calli. We conducted the first deep-sequencing transcriptome profiling of *A. sinensis* under salt stress and observed a large number of differentially expressed genes in response to salinity stress. Moreover, salt stress induced dynamic changes in transcript abundance for novel classes of responsive genes involved in signal transduction, including the genes responsible for hormone signal transduction, receptor-like kinases, MAPK cascades, Ca^2+^ signal transduction, and transcription factors. This study will aid in selecting the target genes to genetically regulate *A. sinensis* salt-stress signal transduction and elucidating the biosynthesis of 2-(2-phenylethyl)chromones under salinity stress.

**Electronic supplementary material:**

The online version of this article (doi:10.1186/s12870-016-0803-7) contains supplementary material, which is available to authorized users.

## Background

*Aquilaria sinensis* is a tropical evergreen tree widely distributed in Fujian, Guangdong, Guangxi and Hainan provinces in China and the other countries such as Vietnam, India, Indonesia, Malaysia, and Thailand [1]. Under stress conditions such as infected by fungi or wounded by wind, lighting, and bited by insects, resin-impregnated heartwoods are slowly forming in the trunk and branches of *A. sinensis* [2–4]. Those resinous heartwoods are commercially called agarwood which has been long-term used as an anti-emetic, digestive, and sedative agent in traditional medicines, and also as incense and peculiar perfume [1]. However, the production of agarwood always takes decades in natural processes, and the natural *Aquilaria* forests have been seriously destroyed in all countries because of the high value and great demand of agarwood. Therefore, *A. sinensis* has been listed in Appendix II of the Convention on Internal Trade in Endangered Species of Wild Fauna and Flora [5]. Under these circumstances, *Aquilaria* trees were cultivated for production of pharmaceutically important and commercially valuble agarwood using artificial methods such as burn-chisel-drill, trunk pruning, and fungi inoculation [3]. However, production of agarwood using artificial methods still takes long time, and the products are always with low quality.

Previous investigations revealed that 2-(2-phenylethyl)chromones are the principal components of argarwood [6–9]. There are more than 100 congeners of 2-(2-phenylethyl)chromones have been reported [10], and many of 2-(2-phenylethyl)chromones have potentially pharmacological activities including neuroprotective activity, cytotoxic activity, antibacterial activity, *AchE* inhibitory, anti-inflammatory activity and antioxidatic activity [7, 11–14]. However, the biosynthesis and regulation of 2-(2-phenylethyl)chromones remains completely unknown.

Agarwood-producing plants are timber species which take a considerably long time to grow and the resinous portion is formed inside of the wood. It makes studies using fresh plants difficult and inconvenient. Thus, establishing calli and cell suspension cultures of *A. sinensis* with high production of 2-(2-phenylethyl)chromones, the principal components of agarwood, would be undoubtedly useful for the studies on the mechanism of agarwood formation [15, 16]. It has been reported that salicylic acid and the crude extracts of fungi could elicit the production of 2-(2-phenylethyl)chromones in the calli and cell suspension cultures of *A. sinensis* [15, 16]. We are, recently, focusing on exploring the mechanism of agarwood formation, establishment of effective method which can be used to induce the production of 2-(2-phenylethyl)chromones in calli and cell suspension is therefore critically important. Surprisingly, we firstly found that salinity stress induced the production of structurally diverse 2-(2-phenylethyl)chromones in *A. sinensis* calli and suspension cells, suggesting that 2-(2phenylethyl)chromones might be responsible to salt stress responses. Identification of these 2-(2-phenylethyl)chromones produced in salt-treated calli and suspension cells would be useful for further research on the biological functions of 2-(2-phenylethyl)chromones in stress responses and the mechanism of agarwood formation.

On the other hand, plants integrate complex signal pathways that may cross-talk and diverge at various steps in response to salt stress [17]. High salinity stress induces the biosynthesis of hormones to regulate the expression of specific genes and metabolites including the most important stress-responsive hormone abscisic acid (ABA) [18]. Salinity stress causes water deficit and osmotic stress, enriching the production of ABA in shoots and roots [19, 20]. The accumulation of ABA can alleviate the inhibitory influence of salinity stress on photosynthesis and growth [21]. Some other phytohormones such as salicylic acid (SA) and brassinosteroids (BR), also participate in plant responses to abiotic stress [22, 23]. Cross-talk among Ca^2+^ signaling pathways and mitogen-activated protein kinase(MAPK) cascades in salt stress responses have been recently been reported [24–26]. Moreover, novel classes of transcription factor family members viral for signal transduction are induced by salt tress, including bZIP, WRKY, AP2/ERF and NAC families which facilitate the expression levels of various genes that eventually influence plant tolerance of salt stresses [27–31]. Previous research indicated that the transcriptional expression of bZIP genes were enriched in salt-sensitive wheat variety under salt stress, but decreased in salt-tolerant cultivar [28]. In *Arabidopsis*, salt stress induced the expression of At WRKY8 which directly binds with the promoter of *RD29A* [29]. Ap2/ERF family members of rice play a significant role in salinity stress response [30]. Over expression of a NAC transcription factor family member in rice and wheat confers salt tolerance [31]. Although traditional forward and genetic approaches can provide valuable insights to salt stress responses, technical limitations may prevent further research. Genome-wide transcriptome analyses have dramatically improved the efficiency of salt stress-related gene discovery [26, 32]. In *Arabidopsis*, more than 20 % of the transcriptome was observed regulating under salinity stress using transcriptome analysis [32]. However, no systematic consensus on the specific classes of genes corresponding to particular signaling events in response to salt stress has been established so far. Identification and characterization of the key factors for salt stress-response signaling pathways will be meaningful for further understanding the mechanism of stress responses and the biosynthesis of specific secondary metabolites.

Herein, NaCl was demonstrated to be an ideal elicitor to induce the production of 2-(2-phenylethyl)chromones in *A. sinensis* calli. Using LC-MS-IT-TOF, 41 phenylethylchromones were identified from NaCl-treated *A. sinensis* calli. In order to elucidate the possible mechanisms of salt stress responses of *A. sinensis*, transcriptome sequencing was performed using Illumina sequencing technology, and the data was analyzed to identify the differentially and specifically expressed transcripts of salt-regulated genes. Concurrently, the novel classes of NaCl-responsive genes relevant to signal transduction in response to salt stress were characterized. The results provided valuable insights for further studies on the mechanism of salt stress signaling transduction and agarwood formation.

## Results and discussion

### Salt stress induced the production of 2-(2-phenylethyl)chromones in *A.sinensis* calli

**Fig 1:**
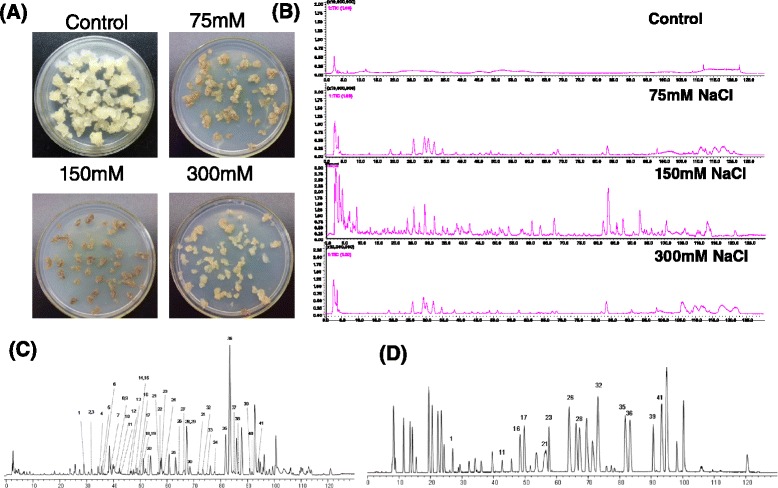
Analysis of 2-(2-phenylethyl)chromones from NaCl-treated *A. sinensis* calli by LC-DAD-IT-TOF-MS system. a Calli treated with different consistence of NaCl at 10dpi. b Effects of treatment with different consistence of NaCl on the production of 2-(2-phenylethyl)chromones. c BPCs of salt-treated *Aquilaria* calli extracts. d BPCs of mixed 2-(2-phenylethyl) chromone standards isolated from agarwood. BPCs: 50–1000 *m/z*

**Fig. 2:**
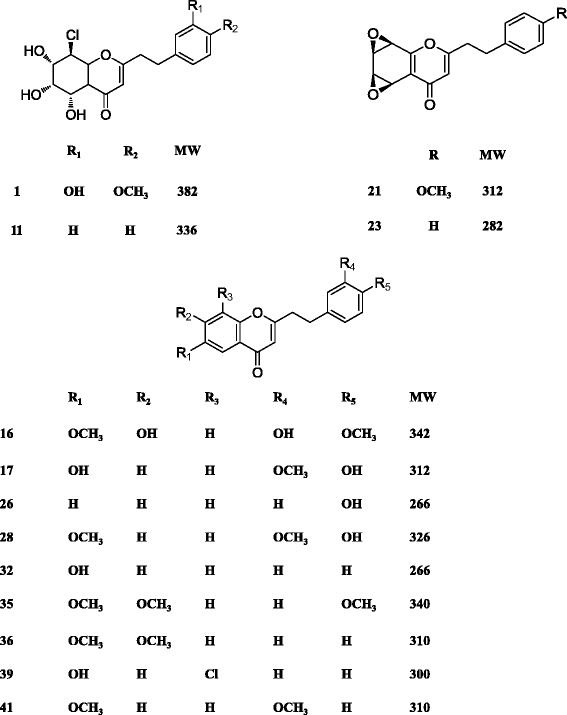
The structures of 2-(2-phenylethyl)chromones identified with the standards

To study the effects of different NaCl concentration on the biosynthesis of 2-(2-phenylethyl)chromones, 75 mM, 150 mM and 300 mM NaCl was applied to the media and 2-(2-phenylethyl)chromones were measured by LC-MS-IT-TOF system at 10 days (Fig. 1a). The peaks of tentative 2-(2-phenylethyl)chromones in the BPC profiles of calli extracts indicated that the most species and contents of 2-(2-phenylethyl)chromones were induced by 150 mM NaCl (Fig. 1b). No 2-(2-phenylethyl)chromones were produced in the control calli (no NaCl supply) (Fig. 1a and b). Our experiments indicated that the accumulation of 2-(2-phenylethyl)chromones kept constantly increasing until four weeks in NaCl-treated calli. Therefore, extracts of *A.sinensis* calli treated with 150 mM NaCl for 4 weeks were analyzed by LCMS-IT-TOF. The BPC profiles of *A.sinensis* calli extracts and mixed standards comprising 33 known 2-(2-phenylethyl)chromones isolated from agarwood are shown in Fig. 1c and d. Forty one 2-(2-phenylethyl)chromones were putatively identified on the basis of their UV and MS data, and 13 of them were unambiguously identified by comparing their retention time (*R*_*t*_) on HPLC chromatogram, UV and MS data with those of authentic compounds. The other 28 compounds were tentatively identified by their predicted molecular formulas deduced from their HRESIMS data, and further confirmed by comparison of their MS/MS data with those of in literature [10]. All the data of 2-(2-phenylethyl)chromones identified from NaCl-treated *A. sinensis* calli are summarized in Table 1, including *R*_*t*_, molecular formula, calculated and experimental molecular weight (*m/z*), error (a relative error between calculated value and measured value) in generated molecular formula, and MS/MS data. The structures of 13 unambiguously identified 2-(2-phenylethyl)chromones from NaCl-treated *A.sinensis* calli are represented in Fig. 2. Previous studies showed that crude extracts of *Melanotus flavolives* (B.etc.) Sing. only induced four 2-(2-phenylethyl)chromones in *A.sinensis* cell suspension cultures [15]. In this study, we firstly used the salt treatment which is the most important abiotic stress to produce a lot of 2-(2-phenylethyl)chromones. These results indicated that salt stress was the effective method to induce the production of 2-(2-phenylethyl)chromones in calli.

**Table 1:** Identified and tentative 2-(2-phenylethyl)chromones compounds from the salt-treated *Aquilaria* calli

Peak number	t_R_ (min)	Molecular formula	*m/z* experimental	*m/z* calculated	Error (ppm)	IT/MS/MS fragment
1^*^	29.03	C_18_H_19_O_7_Cl	383.0913	383.0892	5.48	365, 137
2	32.12	C_18_H_19_O_6_Cl	367.0920	367.0941	−5.72	137
3	32.54	C_18_H_19_O_7_Cl	383.0913	383.0892	5.48	365, 137
4	35.01	C_18_H_19_O_6_Cl	367.0955	367.0943	3.27	137
5	35.87	C_17_H_17_O_5_Cl	337.0821	337.0837	−4.75	319, 195
6	36.59	C_17_H_17_O_6_Cl	353.0820	353.0786	9.63	335
7	39.29	C_17_H_17_O_5_Cl	337.0844	337.0837	2.08	319, 195
8	41.32	C_18_H_16_O_6_	329.1017	329.1020	−0.91	137, 122
9	41.48	C_18_H_20_O_5_	317.1376	317.1384	−2.52	121, 299
10	42.09	C_18_H_18_O_6_	331.1164	331.1176	−3.62	313
11^*^	42.99	C_17_H_17_O_5_Cl	337.0821	337.0837	−4.75	319, 195
12	45.09	C_17_H_14_O_5_	299.0904	299.0914	−3.34	193, 148
13	46.62	C_19_H_18_O_6_	343.1173	343.1176	−0.87	207, 192
14	48.47	C_18_H_16_O_5_	313.1072	313.1071	0.32	206, 191
15	48.99	C_17_H_17_O_5_Cl	337.0844	337.0837	2.08	319, 195
16^*^	49.37	C_19_H_18_O_6_	343.1176	343.1176	0	137
17^*^	50.16	C_18_H_16_O_5_	313.1072	313.1071	0.32	137
18	51.35	C_17_H_17_O_4_Cl	321.0889	321.0888	0.31	303, 212
19	51.77	C_17_H_14_O_4_	283.0965	283.0965	0	192
20	52.89	C_18_H_16_O_5_	313.1071	313.1071	0	137
21^*^	57.51	C_18_H_16_O_5_	313.1071	313.1071	0	121, 192
22	58.06	C_19_H_18_O_4_	327.1227	327.1227	0	220
23^*^	58.48	C_17_H_14_O_4_	283.0965	283.0965	0	192
24	58.98	C_20_H_20_O_6_	357.1339	357.1333	1.68	137, 220
25	63.28	C_18_H_16_O_4_	297.1123	297.1121	0.67	121
26^*^	64.80	C_17_H_14_O_3_	267.1011	267.1016	−1.87	107
27	66.05	C_18_H_15_O_5_Cl	347.0681	347.0681	0	137
28^*^	67.35	C_19_H_18_O_5_	327.1229	327.1227	0.61	137
29	67.78	C_18_H_16_O_4_	297.1126	297.1121	1.68	206, 191
30	69.14	C_18_H_16_O_4_	297.1123	297.1121	0.67	107, 191
31	71.68	C_19_H_18_O_5_	327.1127	327.1127	0	137
32^*^	73.00	C_17_H_14_O_3_	267.1016	267.1016	0	176
33	75.58	C_19_H_18_O_6_	343.1176	343.1176	0	137, 167
34	77.18	C_19_H_16_O_5_	325.1063	325.1071	−2.46	151
35^*^	82.33	C_20_H_20_O_5_	341.1381	341.1384	−0.88	121, 220
36^*^	83.90	C_19_H_18_O_4_	311.1277	311.1278	−0.32	181, 220
37	85.35	C_19_H_18_O_6_	343.1176	343.1176	0	121
38	86.96	C_18_H_16_O_5_	313.1071	313.1071	0	222
39^*^	91.24	C_17_H_13_O_3_Cl	301.0625	301.0626	−0.33	210, 170
40	92.32	C_17_H_14_O_2_	251.1067	251.1067	0	
41^*^	93.55	C_19_H_18_O_4_	311.1277	311.1278	−0.32	121, 190

*: 2-(2-phenylethyl) chromone derivatives identified with standards

(1)8-Chloro-5,6,7-trihydroxy-2-(3-hydroxy-4-methoxyphenethyl)-5,6,7,8-tetrahydro-4H-chromen-4-one; (11) 8-Chloro-2-(2-phenylethyl)-5,6,7-trihydroxy-5,6,7,8-tetrahydrochromone; (16) 7-Hydroxy-6-methoxy-2-[2-(3′-hydroxy-4′-methoxyphenyl)ethyl]chromone; (17) 6-Hydroxy-2-[2-(4′-hydroxy-3′-methoxyphenyl)ethenyl]chromone.; (21) Oxidoagarochromone B; (23) Oxidoagarochromone A; (26) 2-(2-4′- hydroxyphenylethyl)chromone; (28) 6-Methoxy-2-[2-(3-methoxy-4-hydroxyphenyl)ethyl]chromone; (32) AH3: 6-Hydroxy-2-(2-phenylethyl)chromone; (35) AH8: 6,7-Dimethoxy-2-[2-(4-methoxyphenyl)ethyl]chromone; (36) AH6: 6,7-Dimethoxy-2(2-phenylethyl)chromone; (39) 6-Hydroxy-8- chloro −2-(2-phenylethyl) chromone; (41) 6-Methoxy-2-[2-(3′-methoxyphenyl)ethyl]chromone

### Optimization of Illumina sequencing timing

**Fig. 3:**
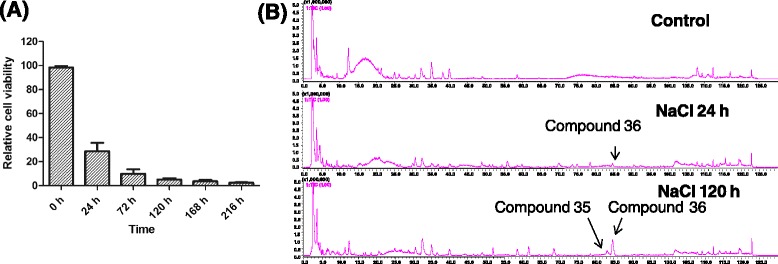
Effects of NaCl treatment on the cell activity of *A. sinensis* calli and production of 2-(2-phenylethyl)chromones at different time points. **a** Relative cell activity of calli exposed to 150 mM NaCl treatment. Values was means standard error (*n* = 3). Means denoted by the same letter did not significantly differ at *P* <0.05 according to Duncan’s multiple range tests. **b** BPCs of 150 mM NaCl treated *A. sinensis* calli extracts at 0 h, 24 h and 120 h

Previous experiments indicated that the cell were almost died at 10 days, in order to determine the best time for tanscriptome analysis, cell activity and 2-(2-phenylethyl)chromone accumulation in calli treated with 150 mM NaCl was investigated at 0 h, 24 h,72 h, 120 h, 168 h and 216 h. The results revealed that the activities of the cells treated with NaCl decreased significantly at 24 h and 120 h, and the activities reached to 30 % and 5 % of the control, respectively (Fig. 3a). We also discovered that the main 2-(2-phenylethyl)chromones detected from the calli were 6,7-dimethoxy-2-[2-(4'-methoxyphenyl)ethyl] chromone (compound **35**) and 6,7-dimethoxy-2-(2-phenylethyl) chromone (compound **36**) during the early inducing period (Fig. 3b). These two compounds are also the main 2-(2-phenylethyl)chromones in agarwood and kept increasing in the wood tissues of *A. sinensis* with the time of fungal infection [27]. Therefore, the occurrence of these two 2-(2-phenylethyl)chromones could be used as an important indicator for studies on the formation of agarwood. Compound **36** was firstly detected in the NaCl-treated calli for 24 h (Fig. 3b), and then the contents of compounds **35** and **36** increased constantly. The production of 2-(2-phenylethyl)chromones **35** and **36** increased remarkably at 120 h (Fig. 3b), continuously, high-quality and sufficient RNA could be isolated from the calli which were treated with 150 mM NaCl until 120 h. Therefore, three cDNA libraries from the control and induced *A.sinensis* calli treated with salt at 24 h and 120 h were constructed using Illumina sequencing.

### Transcriptome sequence assembly and annotation of the unigenes

Three cDNA libraries which were generated with mRNA from control calli, and calli induced by 150 mM NaCl at 24 h and 120 h were assembled and annotated. A total of 68 962 124, 70 631 522 and 70 951 038 clean reads for control (designated control) and induced calli which treated with 150 mM NaCl for 24 h (designated induced-24 h) and 120 h (designated induced-120 h) were generated after removal of the adaptors and unknown or low-quality reads, giving a total of 6 206 591 160 nt, 6 356 836 980 nt and 6 385 593 420 nt for the control, induced-24 h and induced-120 h library (Table 2). After complete assembly of the reads, 104 316, 99 429 and 98 697 contigs with median contig size of 476 nt, 466 nt and 474 nt, were yielded from the control, induced-24 h and induced-120 h library, respectively. Further assembly analysis showed that the control, induced-24 h and induced-120 h library consisted of 91 835, 83 674 and 83 674 unigenes (Table 2), respectively. However, there were 93 041 unigenes with a mean length of 1562 nt were generated from three libraries. The length distribution of unigenes was shown in the Additional file 1: Table S1.

**Table 2:** Summary statistics for sequencing and sequence assembly for three libraries prepared from the control and salt-treated calli

Features	Control	Induced-24 h	Induced-120 h
Total clean Reads	68 962 124	70631522	70951034
Total Clean Nucleotides (nt)	6 206 591 160	6 385 593 420	6 356 836 980
Unigenes	91835	83674	83870
Total Length (nt)	117 782 876	95 610 624	101 385 512
Mean Length (nt)	1283	1143	1209
N50	2269	2062	2166

**Fig. 4:**
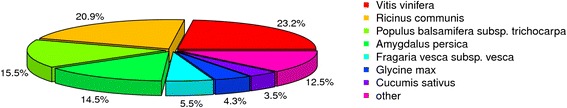
Species distribution of unigenes by BLASTX annotation. The figure indicated species distribution of unigenes BLASTX annotation with a cut-off E-value of 1.0E^5^. Different color showed different species

Functions of the unigenes were annotated by BLASTX based on sequence similarity to sequences in the public databases, including NR, Swiss-Prot, KEGG, COG and GO database, and then aligned to the nucleotide database NT (E-value ≤ 1.0e^−5^) by BLASTN. There were 29 387 unigenes matched to one or more database and a total of 65585 unigenes were annotated. NR classification results revealed that there were 64092 unigenes matched to this database and the unigenes of *A.sinensis* shared 23.2 % and 20.9 % similarity to the homologs with *Vitis vinifera* and *Ricinus communis* by BLASTX annotation, followed 15.5 % and 14.5 % for *Populus balsamifera* and *Amygdalus persica*, respectively (Fig. 4). To further classify the function of the total unigenes, the unigenes were annotated by the GO, COG and KEGG database. GO analysis indicated that the unigenes were grouped into three main categories (biological process, cellular component and molecular function), which together include 55 function classes (Additional file 2: Figure S1; See supporting information). 29395 unigenes had COG annotations and were distributed in 25 clusters including the largest group “General function prediction”, followed by the group of “replication, recombination and repair” and “transcription” (Additional file 3: Figure S2; See supporting information). Gene annotation and pathway mapping in KEGG database indicated that 40552 unigenes were distributed in 128 KEGG pathways. The top three KEGG pathways which include the largest number of unigenes were metabolic pathways, biosynthesis of secondary metabolites and plant-pathogen interaction (Additional file 4: Table S2; See supporting information).

With the advent of sequencing, genome-wide analyses become available in many plant species and have significantly improved the efficiency of gene discovery. However, no genomic data is available for *A.sinensis*. The Illumina technology have been the first choice in field of transcriptome sequencing studies since 2012, owing to the increasing in sequencing length of reads to 150 bp or more [33]. In this study, we carried out *de novo* transcriptome assembly of *A.sinensis* calli. To date, the transcriptome information of *A.sinensis* was acquired by Roche 454 GS platform [2]. However, our *de novo* transcriptome analysis generated greater depth of sequencing, obtaining more complete coverage of the transcriptome comparing with 454 pyrosequencing in previous study. In this study, our transcriptome assembly was compared with the previous published transcriptomes using 454 pyrosequencing [2]. As shown in Additional file 1: Table S1, more than 57 % unigenes were greater than 1 kb, and more than 73 % unigenes were greater than 500 bp. However, over 70 % of the unigenes assembled by 454 GS platform were 200 bp and 600 bp long. These results indicated that our seq-RNA assembly captured larger portion of the transcriptome of *A.sinensis*. Owing to lack of genomic resources for *A.sinensis,* the proportions of unigenes which significantly corresponding to the known proteins in GenBank were considered as another useful metric. Nearly 68.89 % of our unigenes had matched in NR protein database, and this value was higher than 42.8 % reported in 454 GS platform assembly [2]. Thus, the *de novo* assembly of mRNA-seq will significantly improve the genome annotation of *A.sinensis* and be used for further study on the functional members of gene families.

### Functional analysis of differentially expressed genes

**Fig. 5:**
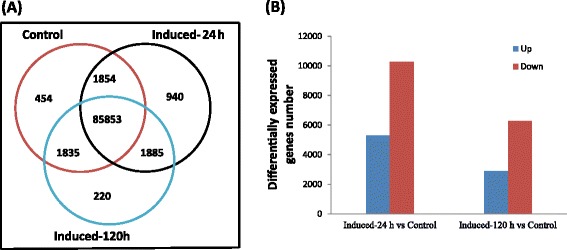
Comparison of the unigenes expressed in the salt-treated and control labraries. **a** Venn diagram showing the unigenes expressed in the control and salt-treated calli. **b** Changes in gene expression profiles among the control, induced-24 h and induced-120 h calli libraries. The number of up-regulated and down-regulated DEGs between the control and induced-24 h, control and induced-120 h libraries were shown. We judged the significance of gene expression difference with the threshold FDR(False Discovery Rate) ≤ 0.001 and the absolute value of log_2_Ratio ≥ 1

To investigate the gene expression changes in response to salinity stress, the gene expression level was measured in fragment per kilobase of exon per million fragments mapped (FRKM) and the false discovery rate [FDR] < 0.001 and the absolute value of log2 Ratio ≥ 1 was used as a threshold to estimate the statistical significance of transcript expression. A total of 18069 differentially expressed genes were identified, including 454, 940 and 220 expressed uniquely in control, induced-24 h and induced-120 h libraries, respectively; 10 881 unigenes were expressed in three libraries but at different levels (Fig. 5a). After induced by salt treatment for 24 h, 5313 genes were induced while 10266 genes were down-regulated. However, 2898 genes were up-regulated and 6268 genes were down-regulated after induced by salt stress for 120 h (Fig. 5b).

**Fig. 6:**
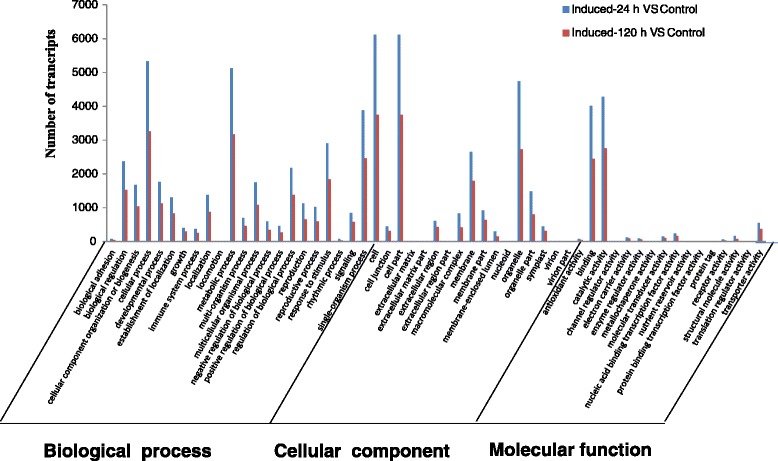
Gene ontology (GO) functional enrichment analysis of differentially expressed genes in salt-treated calli relative to control calli. The functions of DEGs covered three principal categories : biological process, cellular component and molecular function

The differentially expressed genes from the control and induced libraries were organized in functional categories by GO enrichment analysis (Fig. 6). According to the sequence homologies, the differentially expressed genes were assigned to three principal categories: biological process, cellular component and molecular function, which contained 22, 17 and 14 functional groups. Among these groups, the induced-24 h VS control and induced-120 h VS control comparisons had similar distribution of gene functions in biological process, cellular component and molecular function. However, the GO terms “cell periphery”, “extracellular region” and “external encapsulating structure” were significantly enriched after inducing by salt stress for 24 h, whereas “cell periphery”, “anchored to membrane” and “regulation of meristem growth” were primarily enriched after inducing by salt stress for 120 h (Fig. 6). We also found that a high percentage of transcripts after inducing by salt for 24 h and 120 h fell into the functional groups: “cell”, “cell part”, “cellular process”, “metabolic process” and “organelle part”(Fig. 6). To further analysis the function of differentially expressed transcripts, the differentially expressed genes were mapped in the KEGG database. The KEGG analysis indicated that 6621 DEGs with pathway annotation were distributed in 125 KEGG pathways after salt treatment for 24 h, and among these pathways, 31 pathways with P-value ≤ 0.01 were significantly influenced (Additional file 5: Table S3; See supporting information). After inducing 120 h by 150 mM NaCl, 4168 annotated DEGs transport involved in 123 KEGG pathways, and 40 pathways with P-value ≤ 0.01 were significantly enriched (Additional file 6: Table S4; See supporting information). Notably, remarkable enrichment was observed in plant-pathogen interaction pathway, stilbenoid, diarylheptanoid and gingerol biosynthesis pathway, plant hormone signal transduction pathway and pheylpropanoid biosynthesis after inducing by salt for 24 h and 120 h (Table 3). These results suggested that the transcriptome of *A.sinensis* calli was remarkably affected in response to salinity stress and provided resources for screening genes required for salinity stress and agarwood formation.

**Table 3:** Significantly enriched KEGG pathways induced in *A.sinensis* in response to salt stress

	Enriched P-value*	
Pathway category	Induced-24 h VS Control	Induced-120 h VS Control
Plant-pathogen interaction pathway	2.35e-44	1.71e-65
Stilbenoid, diarylheptanoid and gingerol biosynthesis	3.33e-24	1.19e-38
Plant hormone signal transduction	1.15e-21	3.12e-27
Pheylpropanoid biosynthesis	8.90e-20	1.65e-33

### Confirmation of differentially expressed candidate transcripts by quantitative real-time PCR (qRT-PCR)

**Fig. 7:**
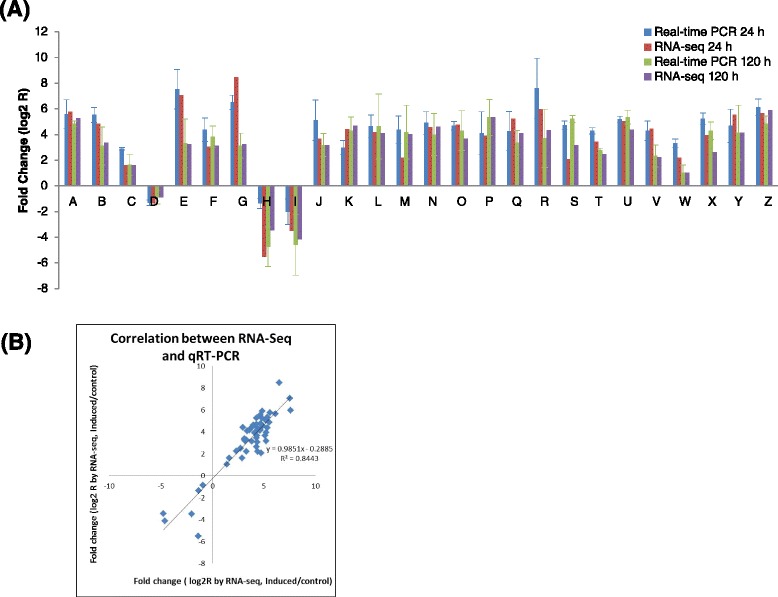
Validation of the relative expression levels of differential expression transcripts by quantitative RT-PCR (qRT-PCR) analysis. **a** Expression profiles of the selected DEGs in the salt-treated calli relative to the control calli, as determined by qRT-PCR(24 h: blue; 120 h:green) and RNA-seq(24 h:red; 120 h: purple). The x-axis indicated the annotation of the selected DEGs. The y-axis indicated the normalized expression level of the genes. A: Calmodulin 1; B:Calcium-binding protein CML37; C: Calcium-dependent protein kinase 10; D: Calcium-dependent protein kinase 13-like; E: Mitogen-activated protein kinase kinase kinase A; F: Mitogen-activated protein kinase kinase kinase 2; G: Mitogen-activated protein kinase kinase kinase 2; H: Auxin influx carrier; I: Auxin response factor 4; J: G-type lectin S-receptor-like serine/threonine-protein kinase; K: Cysteine-rich receptor-like protein kinase 25; L: LRR receptor-like serine/threonine-protein kinase FLS2; M: WRKY transcription factor 75; N: WRKY transcription factor 40; O:WRKY transcription factor 29; P: Ethylene-responsive transcription factor ERF(AP2/ERF); Q: MYB-related protein MYB4; R: MYB superfamily protein 1; S: Methyltransferase PMT15; T: Caffoyl-CoA-*O*-methyltransferase; U: Caffeic acid 3-O-methyltransferase; V: Chalcone synthase; W: Respiratory burst oxidase homolog protein A; X:Respiratory burst oxidase homolog protein B; Y: Respiratory burst oxidase homolog protein D; Z: Pathogenesis-related protein STH-2. The transcriptional level of the selected genes was performed by qRT-PCR with three biological replications and action was used as an internal reference. Error barsrepresent the standard deviations of qRT-PCR signals (*n* ≥ 3). **b** Correlation of the expression ratio of selected DEGs analyzed by qRT-PCR and RNA-seq

To confirm the reliability of the RNA-seq results, a total of 26 candidate genes were selected for qRT-PCR analysis with specific primers (Additional file 7: Table S5; See supporting information). The analysis results suggested that all 26 DEGs selected had the same expression trends as Illumia-Solexa sequencing (Fig. 7a). For example, both qRT-PCR and RNA-seq analysis indicated that the Mitogen-activated protein kinase kinase kinase (MAPKKKA, MAPKKK2 and MAPKKK3), calmodulin, WRKY transcription factors (WRKY39, WRKY40 and WRKY75), caffeoyl-CoA-*O*-methyltransferase, and chalcone synthase 1(CHS1) were significantly more highly expressed in salt-treated calli compared with the control calli. Otherwise, suppression of auxin influx carrier and auxin response factor 4 by salt treatment indicated by RNA-seq analysis was verified by qRT-PCR analysis. Furthermore, a high correlation (R^2^ = 0.8443) was detected between RNA-seq and qRT-PCR (Fig. 7b). These results demonstrated the changes in the gene expression analyzed by RNA-seq reflecting the practical transcriptome difference between the control and salt-treatment calli.

### Salinity stress induced a complex hormone signal pathway

**Fig. 8:**
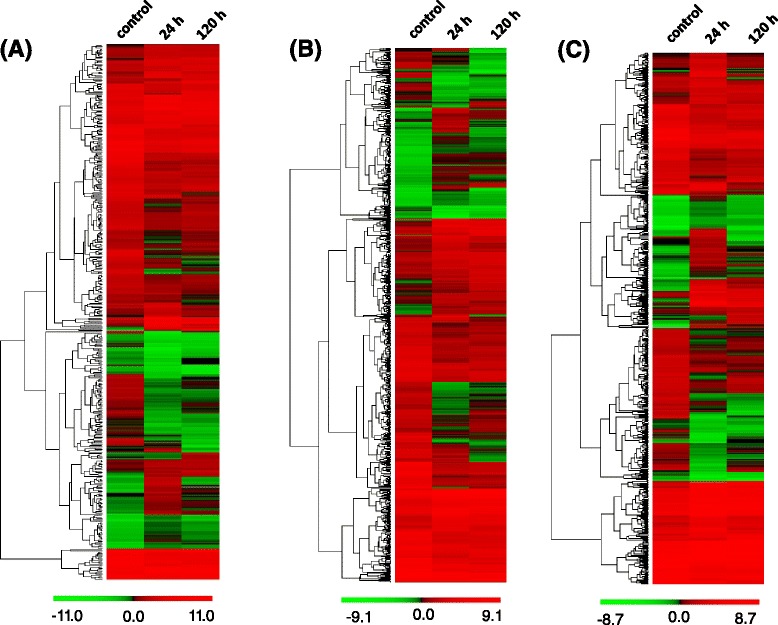
Hierarchical clustering of differentially expressed genes involved in plant hormone signal transduction pathways, receptor-like kinases and transcription factors. Hierarchical clustering of differential expression transcripts among control, induced-24 h and induced-120 h libraries was conducted by the software PermutMatrix v1.93 and based on log_2_(FPKM) data for the intensity of expression of each differentially expressed genes. **a** Herarchial clustering of differential expression profiles related to plant hormone signal transduction pathways. **b** Herarchial clustering of differentially expressed genes annotated as receptor-like kinases. **c** Herarchial clustering of differentially expressed genes annotated as transcription factors. FPKM: numbers of fragments per kilobase of exon per million fragments mapped

Salinity stress in plant induced hormone-independent signaling pathway and hormone biosynthesis [34]. KEGG enrichment analysis showed that 2 042 out of 40 402 *A.sinensis* calli genes annotated as being related to hormone signal transduction pathways were detected in three libraries and 396 of these genes were differentially expressed in calli under salinity stress. Among the hormone signal transduction related DEGs, 130 were up-regulated and 202 down-regulated at 24 h induced by salt treatment, while 74 of genes were up-regulated and 163 were down-regulated at 120 h induced by salt treatment, a total of 175 were co-regulated in 24 h and 120 h for salt stress. Hierarchical clustering of the differentially expressed hormone-related genes indicated overall difference at control, 24 h and 120 h in response to salt stress (Fig. 8a). Pathway enrichment analysis indicated that 8 of hormone-independent signaling pathways including ABA, cytokinine, auxin, brassinosteroid, jasmonic acid, salicylic acid, ethylene and gibberellin pathways were induced in calli after salt treatment for 24 h and 120 h (Table 4). However, more than 70 % of DEGs related to auxin, cytokinine and ethylene pathways were down-regulated, whereas more than 50 % of DEGs required for gibberellin, salicylic acid were up-regulated. These results indicated that salinity stress induced a complex hormone signal transduction pathway.

**Table 4:** The number of differentially expressed genes involved in in the hormone signal transduction pathways

Family, group, or pathway	Number of unigenes					
	Total DEGs	Induced-24 h		Induced-120 h		Co-regulated unigenes
		Up-unigenes	Down-unigenes	Up-unigenes	Down-uigenes	
Abscisic acid	53	19	24	16	19	25
Abscisic acid receptor PYR/PYL family	4	1	2	2	1	2
Protein phosphatase 2C	41	16	17	13	13	18
ABA responsive element binding factor	8	2	5	1	5	5
Cytokinine	79	16	48	2	51	38
Arabidopsis histidine kinase 2/3/4(CRE1)	38	0	35	0	34	31
Histidine-containing phosphotransfer protein (AHP)	2	0	2	0	0	0
Two-component response regulator ARR-B family	34	16	9	2	13	6
Two-component response regulator ARR-A family	5	0	2	0	4	1
Auxin	75	16	47	9	39	41
Auxin influx carrier	7	1	6	1	6	7
Auxin-responsive protein IAA	18	5	12	2	11	11
Transport inhibitor response 1	1	0	1	0	1	0
Auxin response factor(ARF)	31	0	25	0	19	13
Auxin responsive GH3 gene family	6	3	2	3	2	4
SAUR family protein	12	7	4	3	4	6
Brassinoteroid	61	31	23	27	13	31
Protein brassinosteroid insensitive 2(BIN2)	1	1	0	0	0	0
Brassinosteroid resistant 1/2	4	0	4	0	4	4
Xyloglucan:xyloglucosyl transferase TCH4	5	4	1	1	0	0
Cyclin D3	5	5	0	4	0	3
BR-signaling kinase	3	1	2	0	0	0
Brassinosteroid insensitive1-associated receptor kinase 1	41	19	15	21	8	22
Serine/threonine-protein kinase BRI1	2	1	1	1	1	2
Jasmonic acid	36	19	12	7	11	13
Coronatine-insensitive protein 1	1	0	1	0	1	1
Jasmonate ZIM domain-containing protein(JAZ)	9	5	2	4	2	3
Transcription factor MYC2	26	14	9	3	8	9
Salicylic acid	22	15	5	7	3	8
Transcription factor TGA	14	9	5	2	1	3
Pathogenesis-related protein 1	8	6	0	5	2	5
Ethylene	37	10	24	2	13	12
Serine/threonine-protein kinase CTR1	25	1	22	0	11	9
Ethylene-insensitive protein 3	3	1	1	1	1	2
EIN3-binding F-box protein	9	8	1	1	1	1
Gibberellin	33	14	10	8	10	9
Gibberellin receptor GID1	9	6	2	4	1	4
DELLA protein	21	8	6	4	6	3
F-box protein GID2	3	0	2	0	3	2

High salinity elicits rapid and constantly changes in gene expression overlapping with responses to the hormone [34]. Abscisic acid is an essential hormone that mediates plant growth in response to salinity stress through regulating the expression of many genes which encode various proteins vital for biochemical and physiological processes. ABA mediates the salt-stress signaling transduction pathway through the important signaling components, including the ABA receptor PYR/PYL family proteins, the group of protein phosphate 2C, the members of serine/threonine –protein kinase SnRK2, and ABA responsive element binding proteins [35, 36]. In *A.sinensis* calli during salinity stress, the proteins of ABA receptor PYR/PYL family, phosphate 2C family and ABA responsive element binding proteins family showed significantly differential expression patterns, however, the transcripts which annotated as serine/threonine-protein kinase SnRK2 showed no obvious changes(Table 4). The comparative analysis of expression profiles indicated a crosstalk between salt stress and ABA signal pathway. As well as the well-known stress-responsive ABA, other phytohormones were also responsible for salt stress. Recent studies indicated that alterations in cytokinine signaling influenced plant response to abiotic stress, seed germination, cell division, and floral initiation [37, 38]. In *Arabidopsis*, endogenous cytokinin overproduction decreased salt stress resistance, however, low cytokinin levels promoted salt-induced senescence in tomato [39]. Our transcriptome analysis indicated that the majority of receptor kinases and response regulators responsible for cytokinine signal transduction were significantly decreased under salt treatment (Table 4). For example, the majority of changed *A.sinensis* CRE1 genes which are the important cytokinin receptors were down-regulated under salt stress. Down-stream genes such as ARR-B genes indicated both up- or down-regulated under salt stress at different time point. Plants utilized auxin signaling to modify root development when responding to diverse biotic and abiotic signals including salinity stress [37, 38]. In *Arabidopsis,* salt stress inhibits the PIN2 (an auxin efflux carriers) expression which is known to dominate gravitropic root response by monitoring bicipital auxin transport [40]. A total of 72 genes required for the auxin signal pathway were differentially expressed under salt treatment in calli. The majority of changed *Aquilaria* auxin influx carrier and auxin response factor genes were down-regulated under salt stress conditions in calli, however, the genes annotated as auxin-responsive protein (IAA), auxin responsive GH3 gene family and SAUR family protein were both up- and down- regulated during salt treatment (Table 4). In addition, many *Aquilaria* brassinoteroid signal transduction genes, such as brassinoteroid resistant1/2 and brassinoteroid insensitive 1-associated receptor kinases (BAK1) family of genes, were also differently expressed under salt stress. In particular, the brassinoteroid resistant1/2 family genes were down-regulated during salt treatment, however, the BAK1 family members which acting on serine/threonine containing substrate and required for brassinosteroid signal transduction [41], were both up- and down-regulated during salt stress in calli at different time point (Table 4). Furthermore, families of jasmonate ZIM domain-containing protein (JAZ), transcription factor MYC2 and TGA which involved in jasmonic acid and salicylic acid signal transduction [42], respectively, were also different expressed under salt stress in calli, with majority up-regulated. Besides ABA, cytokinine, auxin, brassinoteroid, jasmonic acid and salicylic acid signal pathways in response to salinity stress, other hormones including ethylene and gibberellin also play an important role for salt stress signaling transduction. The comparative analysis of expression profiles indicated that salinity stress induced complex hormone signal pathways.

### Salt stress regulated the genes associated with signal transduction: receptor-like kinases, Ca^2+^ -binding proteins and MAPK proteins

An appropriate defense response to salt stress initially requires recognition of the stress. One of the largest groups of receptor proteins related to the responses to various biotic and abiotic stresses is the receptor-like kinase superfamily which possesses an amino-terminal signal sequence, a transmembrane segment and intracellular serine/threonine kinase domain [43–45]. We identified 688 DEGs annotated as receptor-like kinases induced in the calli under salinity stress (Additional file 8: Table S6, See supporting information). A total of 525 DEGs identified at 24 h in calli after induced by salt stress were annotated as receptor-like kinases, while a total of 409 DEGs categorized as receptor-like kinases were induced by salt treatment at 120 h. Among these DEGs, 265 were up-regulated and 263 were down-regulated induced by salt treatment after 24 h, whereas, 181 were up-regulated and 230 were down-regulated at 120 h after induced by salt, however, with 279 DEGs co-regulated in 24 h and 120 h after induced by salt. Hierarchical clustering of the differentially expressed genes annotated as receptor-like kinases suggested overall difference at control, 24 h and 120 h in the calli induced by salinity stress (Fig. 8b).

At least four receptor-like kinase families have been reported to be induced in stress-responsive genes: LRR receptor-like serine/threonine-protein kinase (LRR-RLK), proline-rich receptor-like kinase (PERK), cysteine-rich receptor-like protein kinase (CRR-RLK), and G-type lectin S-receptor-like serine/threonine-protein kinase(SRK) (Additional file 8: Table S6, See supporting information). LRR-RLK family constitutes the largest group among the receptor-like kinases, and regulates various plant processes of plant growth and development underlying the abiotic stress and biotic stress response [44–46]. For instance, FLS2 which is the member of LRR-RLK, recognizes the bacterial flagellin fragment and mediates defense responses in plants [47]. Brassinosteroid insensitive 1-associated receptor kinase (BRI) which is a member of LRR-RLK and is responsible for BR hormones, regulate plant tolerance to abiotic and biotic stress [46]. Consistent with these, we observed that a total of 262 differential expressed transcripts were annotated as LRR-RLK family members, including 88 of FLS2 subgroup members and 16 of BRI subgroup members (Additional file 8: Table S6, See supporting information). For instance, a FLS2 subfamily member, were induced more than 4-fold by NaCl at 24 h and 120 h in calli, and we confirmed this by qRT-PCR. PERK family members act as sensors/receptors in cell wall and monitor changes to the cell wall during plant exposure to abiotic and biotic stresses [48]. The first characterized PERK member was *Brassica napus PERK1*, which expressed in different tissues and rapidly induced by wounding [49]. Another member of PERK family, PERK4, was a positive regulator in ABA and salt stress reponses. We observed that 92 of transcripts for PERK family members detected were regulated in response to NaCl treatment and about half of transcripts were up-regulated. However, we observed the majority of the CRR-RLK and SRK family members were up-regulated under salt stress in different time point, as expected for these two families of stress-inducible receptor-like kinases(Additional file 8: Table S6, See supporting information). Many of other NaCl-responsive receptor-like kinases shown in the table are potentially regulators of the NaCl-stress response in *A.thaliana*. These results suggested that the transcription of the majority of receptor-like genes is controlled and modulated by salt stress, implying their important roles in salt stresses signaling transduction. The downstream events of receptor-like kinase signaling relate to protein phosphorylation via a mitogen-activated protein kinase (MAPK, MAPKK, MAPKKK) cascade [50, 51]. In our analysis, salt stress influenced the expression pattern of several mitogen-activated protein kinase genes. A total of 26 differentially expressed transcripts involved in MAPK pathways and up-regulated transcripts were especially predominant in the mitogen-activated protein kinase kinase kinase family (Additional file 8: Table S6, See supporting information). For instance, three mitogen activated protein kinase kinase kinases that encoded MAPKKKA, MAPKKK2 and MAPKKK3 were transcriptionally enriched (Unigene19214_All, Unigene3063_All, CL5738.contig1_All) at 24 h and 120 h under salt treatment (Table 5), and we conformed this by qRT-PCR. However, most members of mitogen activated protein kinase kinase and mitogen activated protein kinase genes including the protein encode MPK3 and MEKK2 in *A.thaliana* that play an important role for stresses response were down-regulated at two time point[51, 52] (Table 5). Calcium ions (Ca^2+^) have been demonstrated as a second messenger in plant signaling pathways including response to salt stress. It has been reported that salt treatment trigger a transient increase in Ca^2+^ level [53]. The activation of MAPK through stresses is always dependent on binding of Ca^2+^-binding proteins including calmodulins (CaM), calcium-binding protein(CML), calcium-dependent protein kinases (CDPKs) and calcineurin B-like protein(CBL) (Additional file 8: Table S6, See supporting information) [53–56]. In our analysis, 81 differentially expressed unigenes which annotated as Ca^2+^ dependent proteins were responsive to salt stress signal transduction and up-regulated transcripts were especially predominant in CML proteins at 24 h and 120 h under salt stress. *Aquilaria* calli genes encoding Ca^2+^ dependent proteins including CaM1, CML37, CML27, CML29 and CDPK1 which are responsible for plant innate immunity [54–56] (Table 5), showed a high expression, suggesting these proteins might be a component of Ca^2+^ signaling to modulate plant defense response against salt stress. Thus, receptor-like kinases, MAPK cascades and Ca^2+^ dependent proteins involved in salt stress signal transduction.

**Table 5:** Expression patterns of unigenes annotated as members of MAPK cascades and Ca^2+^ signal pathways

Gene ID	Annotation	Induced-24 h		Induced-120 h	
		FC*	P-value	FC	P-value
Unigene19214_All	Mitogen-activated protein kinase kinase kinase A-like MAPKKKA	7.06	0	3.26	0
Unigene3063_All	Mitogen-activated protein kinase kinase kinase 2 MAPKKK2	8.47	0	3.64	6.27E-09
CL5738.Contig1_All	Mitogen-activated protein kinase kinase kinase 3 MAPKKK3	3.07	5.38E-54	3.13	2.58E-56
Unigene21616_All	Mitogen-activated protein kinase kinase 2 MEKK2	−2.50	1.05E-130	−1.41	2.20E-59
CL7087.Contig2_All	Mitogen-activated protein kinase kinase 6 MAPKK6	−2.06	1.68E-08	−1.67	1.56E-06
Unigene2000_All	Mitogen-activated protein kinase 9-like MPK9	−2.60	0	−1.90	2.03E-302
CL9084.Contig2_All	Mitogen-activated protein kinase 3 MPK3	−2.05	7.15E-30	−2.12	3.65E-30
Unigene26936_All	Calmodulin 1 CaM1	5.76	1.29E-60	5.26	5.50E-41
Unigene27810_All	Calcium-binding protein CML37	4.86	0	3.36	1.08E-101
CL4994.Contig1_All	Calcium-binding protein CML27	4.14	0	3.20	0
Unigene11150_All	Calcium-binding protein CML29	3.69	3.56E-42	2.53	8.53E-14
Unigene27873_All	Calcium-dependent protein kinase 1 CDPK1	2.96	1.28-108	3.24	1.56E-141

*: Fold change

### Salt stress regulated the expression of transcription factors genes in calli

Transcription factors are essential for regulation of gene expression, biotic and abiotic stress responses, and signal transduction. The *Arabidopsis* genome possesses at least 1819 predicted transcription factors that have been classified into 56 families. However, there were 428 predicted transcription factors were regulated by salt treatment. In *A.sinensis*, a total of 598 DEGs were annotated as transcription factors. 512 DEGs were induced by 150 mM NaCl at 24 h, with 264 DEGs were up-regulated and 248 DEGs were down-regulated, whereas 330 DEGs were induced by NaCl at 120 h, 128 were up-regulated and 202 were down-regulated, however, only 220 DEGs were co-regulated at 24 h and 120 h (Additional file 9: Table S7, See supporting information). Hierarchical clustering of DEGs annotated as transcription factors suggested overall difference in control, induced-24 h and induced-120 h libraries (Fig. 8c). At least seven transcription factor families have been reported to be enriched in stress-responsive genes: AP2/ERF(Apetala-2/EREBP), MYB(Myeloblastosis), WRKY(named after the wrky amino acid motif), bHLH(basic helix-loop-helix), HOX(Homeodomain-containing transcription factor), NAC(NAC domain protein), and HSF(Heat Shock Factor) (Table 6). AP2/ERF is one of the largest transcription factor family involved in the response to salt stress, which can be further classified into four subfamilies: ERF, DREB, AP2, RAV [57]. Of these, *ERFs* and *AP2s* expression levels were found to be correlated with salt tolerance and activate the downstream salt-responsive genes. In *A.sinensis*, we observed 73 of AP2/ERF family transcripts were regulated by salt stress (Additional file 9: Table S7, See supporting information), and the up-regulated transcripts were detected in three AP2/ERF family’s subgroups(AP2, ERF, DREB) at two time point (Table 6). However, the ERF subfamily members were found to be significantly induced by salt stress at 24 h and 120 h in *A.sinensis* calli (Table 6). Recent analysis of the MYB family members expression level concluded that most of MYB-transcription factor are responsible for stresses and hormones [58]. Consistent with this analysis, in *Arabidopsis*, at least one third of the 84 predicted MYB transcription factors were enriched by NaCl at the transcript abundance level, and in wheat 16 of 60 detectable MYB family members were induced by high salt at transcript level [32, 59]. Similarly, we observed that a total of 71 DEGs were annotated as MYB family members and 14 of 24 co-regulated transcripts were enriched by NaCl (Additional file 9: Table S7, See supporting information). *MYB1*(Unigene9921_All) and *MYB4*(CL3573.Contig1_All), were induced more than 4-fold by NaCl at each of two time point (Table 6), and we confirmed this by qRT-PCR. In addition, the other MYB family members such as *MYB75*, *MYB39*, *MYB78* and *MYB21* were up-regulated by NaCl. The majority of WRKY family members which contain zinc-finger motifs are known to involve in biotic and abiotic response [60]. In *Arabidopsis*, more than 1/2 WRKY transcription factors were induced by NaCl, and the members of *WRKY17*, *WRKY25* and *WRKY33* were significantly enriched [32]. We observed that 58 DEGs were annotated as WRKY family transcription factors and the majority of the WRKY family members were up-regulated by salt treatment at 24 h and 120 h (Additional file 9: Table S7, See supporting information). We also used qRT-PCR to confirm these observations for *WRKY29*, *WRKY40* and *WRKY75* (Table 6), which were significantly enriched by salt stress. Three other large families of transcription factors with distinct DNA binding motifs are bHLH, HOX and NAC genes [32]. All of these families contained NaCl-responsive genes, however, the members of HOX and NAC families were remarkably enriched in NaCl-induced transcripts. Furthermore, most of the HSF family members were enriched by salt stress [61] (Table 6), as expected for this family of stress-responsive transcription factors. Many other transcription families including MYC, GRAS, TGA, TCP and GATA (Additional file 9: Table S7, See supporting information), contained NaCl-responsive genes and played important role for the physiological response to NaCl treatment. The large number of up- or down-regulated transcription factor genes we observed, is consistent with the existence of a complex signal transduction network underlying the response to salt stress.

**Table 6:** Transcription factors co-up-regulated by salt stress at different time in *A.sinensis* calli

Gene ID	Annotation	TF family	Log2 ratio	
			24 h vs control	120 h vs control
Unigene18058_All	Ethylene-responsive transcription factor	AP2/ERF	6.12	4.33
Unigene14856_All	Ethylene-responsive transcription factor ERF109-like	AP2/ERF	4.24	2.55
Unigene7108_All	Ethylene-responsive transcription factor ERF109	AP2/ERF	4.18	2.50
Unigene4181_All	AP2 domain-containing transcription factor	AP2/ERF	3.96	3.67
Unigene29855_All	AP2 domain class transcription factor	AP2/ERF	3.91	5.33
Unigene21646_All	Ethylene-responsive transcription factor ABR1	AP2/ERF	3.06	2.40
Unigene18948_All	Ethylene-responsive transcription factor 1B	AP2/ERF	3.15	1.25
CL248.Contig1_All	Ethylene-responsive transcription factor RAP2-7	AP2/ERF	2.82	1.90
Unigene17032_All	Ethylene-responsive transcription factor 5	AP2/ERF	2.35	1.51
Unigene6220_All	dehydration-responsive element binding protein 2 (DREB2)	AP2/ERF	1.98	1.72
Unigene381_All	Ethylene-responsive transcription factor ABR1	AP2/ERF	1.93	2.14
Unigene8092_All	AP2/ERF domain-containing transcription factor	AP2/ERF	1.84	1.95
Unigene20214_All	ethylene-responsive element binding protein 1	AP2/ERF	1.66	1.25
Unigene14338_All	AP2/EREBP transcription factor	AP2/ERF	1.47	1.46
Unigene25279_All	AP2/ERF domain-containing transcription factor	AP2/ERF	1.40	1.90
Unigene9921_All	MYB superfamily protein 1	MYB	5.95	4.32
Unigene11776_All	Transcription factor MYB75	MYB	5.73	3.60
Unigene14868_All	Transcription factor MYB39	MYB	5.43	3.08
Unigene26918_All	Putative MYB family transcription factor	MYB	5.27	3.86
CL3573.Contig1_All	MYB-related protein MYB4	MYB	5.25	4.09
Unigene28480_All	MYB-related protein 78	MYB	5.09	2.99
Unigene13545_All	Putative MYB family transcription factor	MYB	4.96	2.16
CL5989.Contig1_All	Transcription factor MYB21	MYB	4.71	2.93
Unigene5251_All	MYB-related protein	MYB	4.14	3.10
CL5340.Contig1_All	Putative MYB family transcription factor	MYB	3.81	2.79
CL4273.Contig2_All	MYB-related protein	MYB	2.92	3.11
CL277.Contig1_All	MYB superfamily proteins	MYB	2.08	4.09
Unigene23227_All	myb-related protein	MYB	2.74	1.95
Unigene10979_All	Transcription factor MYB21	MYB	2.37	1.24
Unigene5227_All	myb-related protein 306-like	MYB	1.78	1.21
CL8451.Contig1_All	MYB transcription factor R3 type	MYB	1.58	1.33
Unigene38347_All	WRKY transcription factor 22-like	WRKY	4.79	3.01
CL9188.Contig2_All	WRKY29-1 transcription factor	WRKY	4.75	3.66
Unigene2832_All	Transcription factor WRKY40	WRKY	4.56	4.60
Unigene29386_All	WRKY transcription factor 28	WRKY	3.57	2.40
Unigene27408_All	WRKY transcription factor 23	WRKY	3.07	1.46
Unigene1977_All	WRKY transcription factor 15	WRKY	2.85	1.82
Unigene26943_All	WRKY transcription factor 23	WRKY	2.82	1.94
Unigene4489_All	WRKY transcription factor 47	WRKY	2.58	3.19
CL1269.Contig1_All	WRKY transcription factor 33	WRKY	2.38	2.26
Unigene7236_All	WRKY transcription factor 75	WRKY	2.20	4.03
Unigene23162_All	Probable WRKY transcription factor 14	WRKY	1.99	1.88
Unigene27807_All	WRKY transcription factor	WRKY	1.88	2.40
Unigene20429_All	WRKY transcription factor 17	WRKY	1.82	1.18
Unigene15555_All	WRKY transcription factor 6	WRKY	1.63	1.89
Unigene25162_All	WRKY transcription factor 11	WRKY	1.60	1.28
Unigene11168_All	WRKY transcription factor 65	WRKY	1.28	1.77
Unigene14452_All	WRKY transcription factor 28	WRKY	1.26	1.22
CL8216.Contig1_All	Transcription factor bHLH122	bHLH	2.49	1.28
CL1535.Contig1_All	transcription factor bHLH123	bHLH	1.79	1.14
Unigene29356_All	transcription factor bHLH30-like	bHLH	1.75	1.80
Unigene19013_All	Transcription factor bHLH130	bHLH	1.43	1.39
Unigene25168_All	Homeobox-leucine zipper protein HOX11	HOX	6.31	5.99
Unigene11236_All	homeobox-leucine zipper protein HOX6-like	HOX	4.89	1.89
Unigene15687_All	Homeobox-leucine zipper protein HOX27	HOX	1.85	1.51
Unigene35957_All	homeobox-leucine zipper protein HAT14-like	HOX	1.74	1.71
Unigene27798_All	NAC domain protein NAC4	NAC	6.41	4.04
Unigene24899_All	NAC transcription factor NAM-2	NAC	4.51	1.86
CL6345.Contig1_All	NAC domain-containing protein 68	NAC	2.00	1.35
Unigene14392_All	NAC domain-containing protein 18	NAC	1.88	1.37
Unigene26036_All	NAC domain protein NAC2	NAC	1.39	1.17
CL598.Contig8_All	Heat stress transcription factor	HSF	4.92	5.77
Unigene13160_All	heat stress transcription factor A-4a	HSF	2.20	1.45
CL2103.Contig2_All	Heat stress transcription factor B-2a	HSF	1.95	1.25
Unigene10292_All	Heat stress transcription factor B-2a-like	HSF	1.59	1.10

### Putative genes involved in biosynthesis of 2-(2-phenylethyl)chromones in calli under salinity stress

Our study showed that salinity stress induced the production of 2-(2-phenylethyl)chromones in *A.sinensis* calli. These results suggested that the activities of enzymes which are responsible for biosynthesis of 2-(2-phenylethyl)chromones were enriched by salinity stress. The RNA-seq analysis indicated that the diarylheptanoid and ginerol biosynthesis pathways were significantly enriched, however, curcumin synthase which is a plant polyketide synthase and is the most important synthase for diarylheptanoid and ginerol biosynthesis [62], could not be annotated the three libraries including control, induced-24 h and induced-120 h. According to the structure of 2-(2-phenylethyl)chromones, we proposed that the biosynthesis of 2-(2-phenylethyl)chromones might be similar to diarylheptanoids, and polyketide synthases (PKSs) except curcumin synthase may play a vital role in the biosynthesis of 2-(2-phenylethyl)chromones [63]. The transcriptome data indicated that the expression of chalcone synthase which is a member of type III polyketide synthase was affected after induced by salt for 24 h and 120 h (Additional file 10: Table S8, See supporting information). Among these genes, the chalcone synthase 1 (CHS1) was up-regulated in calli after induced for 24 h and 120 h; however, the expression of CHS2 was reduced at 120 h (Table 7). The phylogenetic tree was constructed according to the PKSs amino acid sequences by Neighbor-Joining method using MEGA 6.0 software to investigate the evolutionary relationship and distribution among PKSs from different species. The phylogenetic tree analysis allow the classification of PKSs in higher plants in two different clusters called CHS(chalcone-prodcuing)and non-CHS (nonchalcone-producing) cluster, and AsCHS1 was grouped in non-CHS cluster, including curcuminoid synthase (CUS) from monocots *Oriza sativa*, diketide-CoA synthase (DCS) and curcumin synthase (CURS) from monocots *Curcuma longa*, and AsCHS2 was grouped in CHS cluster [63] (Additional file 11: Figure S3). These results suggested that AsCHS1 might possess an unusual catalytic potential and could be the important enzyme for biosynthesis of 2-(2-phenylethyl)chromones. 2-(2-phenylethyl)chromones always display *O-*methyl groups on the phenylethylchromone scaffold. It suggested that *O*-methyltransferase plays a critical role in transferring *O*-methyl groups to 2-(2-phenylethyl)chromone scaffolds. Recently, transcriptome analysis result from the medical plant *Glaucium flavum* indicated that the *O*-methyltransferase (OMTs) were required for benzylisoquinoline alkaloid (which displays four *O*-methyl groups at C6, C7, C3’and C4’ on the benzylisoquinoline scaffold) biosynthesis [64]. Transcriptome resource analysis of *Aquilaria* calli revealed the biosynthesis of 2-(2-phenylethyl)chromones required the members of flavonol 3-*O*-methyltransferase and caffeoyl-CoA *O*-methyltransferase families transfer *O*-methyl groups to the 2-(2-phenylethyl)chromones scaffolds (Table 7). RNA-seq analysis showed that a total of 21 DEGs were annotated as flavonol 3-*O*-methyltransferase and a total of 8 DEGs were annotated as caffeoyl-CoA *O*-methyltransferase (Additional file 10: Table S8, See supporting information). More than half of transcripts annotated as *O*-methyltransferase were enriched by salinity stress at 24 h and 120 h. These results demonstrated that chalcone synthase and *O*-methyltransferase play a critical role in biosynthesis of 2-(2-phenylethyl)chromones.

**Table 7:** Expression patterns of genes involved in biosynthesis of 2-(2-phenylethyl)chromones

Gene ID	Annotation	Induced-24 h		Induced-120 h	
		FC*	P-value	FC	P-value
CL6258.Contig1_All	Chalcone synthase (CHS1)	4.43	0	2.23	6.31E-72
Unigene9694_All	Chalcone synthase (CHS2)	--	--	−1.86	0
CL6576.Contig1_All	Flavonol -*O*-methyltransferase-like protein (OMT1)	5.04	0	4.39	0
Unigene30708_All	Flavonol -*O*-methyltransferase 3	4.65	2.41E-94	3.12	8.68E-25
Unigene11142_All	Caffeoyl-CoA-*O*-methyltransferase	3.43	0	2.47	0

*: Fold change

## Conclusions

We firstly identified 41 2-(2-phenylethyl)chromones from the salt-treated *A.sinensis* calli by LC-MS system. To find the transcripts responsible salinity stress, a comprehensive transcriptome analysis was conducted from the control calli and induced calli with 150 mM NaCl treatment. A total of 93 041 unigenes with an average length of 1562 nt were obtained, and they were annotated by comparing them with the public database including NR, Swiss-Prot, KEGG, COG and GO database. Our transcriptional analysis revealed numerous genes that were differentially expressed at 24 h and 120 h under salt stress in calli. The differentially expressed candidate genes from RNA-seq were confirmed by qRT-PCR analysis. We found that numerous genes involved in hormone signal transduction, MAPK cascades signal transduction, Ca^2+^ signal transduction, or encoding receptor-like kinase and transcription factors showed different expressions between the control and salt-treated calli. These differences suggested that a complex signal pathway were induced in response to salinity stress. In addition, our RNA-seq analysis observed that chalcone synthase and *O*-methyltransferase may regulate the biosynthesis of 2-(2-phenylethyl)chromones. These results provide a better understanding of salt stresses signal transduction in *A.sinensis* calli, and clues to facilitate the mechanism of agarwood formation.

## Methods

### Plant material and chemical treatment

The *A.sinensis* leaves(Zhongshan, Guangdong province, China) were cut into 1 cm diameter, surface-sterilized by Clorox (2.5 %) for 10 min, disinfected using 70 % ethanol for 30 s, and washed with sterile distilled water for 4 times. The treated leaves pieces were plated on the MS medium with 2 μg/mL naphthalene-1-acetic acid (NAA) and 1 μg/mL 6-benzylaminopurine (6-BA) for inducing callus. After incubation at 25 °C for one month in dark, callus were sub-cultured onto the fresh MS medium containing 2 μg/mL NAA, 1 μg/mL 6-BA, 1 μg/mL dichlorophenoxyacetic acid (2,4-D) and 1 μg/mg kinetin(KT) every month. To identify and speculate 2-(2-phenylethyl)chromones, the calli were transferred to the medium containing 150 mM NaCl and harvested every 4 weeks for analysis by LCMS-IT-TOF. To optimize the sequencing timing, 150 mM NaCl treated calli were harvested at 0 h, 24 h, 72 h, 120 h, 168 h, and 216 h. For salt treatment, NaCl was added to the medium with a final concentration of 75 mM, 150 mM, and 300 mM. The calli subcultured for 4 weeks after inoculation were transferred onto these medium with or without NaCl, and were harvested at 10 days. Following salt treatment, the calli were collected and washed for 5 min by distilled water.

### Analysis of 2-(2-phenylethyl)chromones of treated *Aquilaria* calli

The calli were dried at 65 °C and extracted with 95 % methanol (0.1 g dried calli with 1 mL 95 % methanol) at room temperature in an ultrasonic bath for half an hour. The extracts were then centrifuged at 12 000 rpm for one hour at 4 °C and the supernatant were analyzed with LC-DAD-IT-TOF-MS system (Shimadzu, Kyoto, Japan). For liquid Chromatography analysis, Chromatographic separations were carried out on an Agilent SB-C_18_ column (250 × 4.6 mm i.d., particle size 5 μm, Agilent Technologies. Palo Alto, CA, USA). The injection volume was set at 10 μL. The mobile phase was composed of acetonitrile (A) and 0.1 % aqueous formic acid (B), and delivered at 1.0 mL/min following the gradient program: 0 – 20 min, 10 % – 20 % A; 20 – 35 min, 20 % – 25 % A; 35 – 55 min, 25 % – 35 % A; 55 – 70 min, 35 % – 38 % A; 70 – 90 min, 38 % – 50 % A; 90 – 105 min, 50 – 70 % A; 105 – 120 min, isocratic 70 % A; 120 – 130 min, 70 % – 90 % A. UV absorption over 190–400 nm was recorded by DAD module. In mass spectrometer domain, an hybrid ion trap-time-of-flight mass spectrometer (IT-TOF-MS, Shimadzu) equipped with an electrospray ionization (ESI) interface was connected to LC system via a PEEK tube (0.13 mm i.d.) to perform high-resolution tandem mass spectrometry. The accurate ion axis was calibrated using the sodium trifluoroacetate (TFA) clusters as reference. Positive mass spectra were recorded in the full scan and automatic multiple stage fragmentation scan modes over a range of *m/z* 100 – 1000 for all MS1, MS2 spectra acquisition. The accuracy of the assigned chemical formula was determined using mass difference tolerance of ± 5 ppm, which was calculated by the deviation between the experimental mass and calculated mass.

### RNA extraction and cDNA library preparation

Total RNA was extracted from the control and NaCl-induced calli at 24 h, 120 h, using the total RNA extraction kit (Norgen, Cat 72200) according to the manufacturer’s recommendations. The RNA concentration and integrity were analyzed by Nanodrop2000 (ND-100 Spectrophotometer, Peqlab). The RNA from the induced calli (three replicates) was mixed in equal proportion to generate the pool for the induced library, and the RNA from the control callus (three replicates from control calli at 24 h and three replicates from control calli at 120 h) was mixed to a pool for the control library.

### Library construction and *De novo* transcriptome sequencing

Total RNA from control and induced calli was purified using oligo(dT) magnetic beads and fragmented into small pieces according to the manufacturer’s instruction (Illumina, San Diego, CA USA). The cleaved fragments were then used to synthesize first-strand cDNA with random hexamer primiers and reverse transcriptase (Invitrogen, Carlsbad, CA USA). Second-strand cDNA was synthesized using DNA polymerase I (Invitrogen, Carlsbad, CA USA), dNTPs and RNase H. Following, the cDNA fragment were amplified by PCR and purified after removal of the ligation adaptors and end-repair to construct the final library. Three libraries with the insert size ranging from 150 to 250 bp were sequenced on the Illumina HiSeq-2000 platform using the 100 bp paired-end approach. In total, 74,881,134, 80,382,270 and 77,355,892 raw reads were generated by Solexa/Illumina sequencing in three libraries respectively, which had been deposited in Sequence Read Archive database in NCBI, Accession No. SRA319923.

### *De novo* Transcriptome Assembly and Annotation of Unigene Functions

After removal of the adaptor sequences, reads in low quality (more than 20%of reads having quality value ≤ 10) and reads with unknown nucleotides larger than 5 %, the clean reads assembly was carried out with short reads assembling program- Trinity program [65]. Firstly, Trinity assembled the reads with a certain length of overlap into contigs, clustered contigs into clusters and constructed de Brujin graphs for each cluster which represented the full transcriptional complexity for a given gene, and then partitioned the full read set among these graphs. Finally, Trinity processed the individual graphs, traced the paths that the reads took within the graph, ultimately programed full-length transcripts, and tested apart transcripts that corresponded to paralogous genes. The final sequences assembled by trinity were defined as ‘unigenes’. The Trinity unigenes of three libraries were further assembled with TGICL software to get non-redundant unigenes for further analysis[66]. The functions of the unigenes were aligned using BLASTX searches (with an E-value threshold of 10^−5^) to the public protein database including the NCBI non-redundant database (NR) (http://www.ncbi.nlm.nih.gov), the Swiss-Pot database (http://www.expasy.ch/spot), Cluster of Orthologous Groups(COG) database (http://www.ncbi.nlm.gov/COG), and Kyoto Encyclopedia of Genes and Genomes (KEGG) database (http://www.genome.jp/kegg). The proteins with highest sequence similarity were retrieved to analysis. COG matched each annotated sequence to an ancient conserved domain to predict and classify possible functions, while KEGG produced the inner-cell metabolic pathways. Based on NR annotation, GO functional annotation and further functional classification were carried out with Blast2GO program (http://www.blast2go.com/b2ghome) [67].

### Functional Analysis of differentially expressed genes

The unigenes expressed value and transcript levels were calculated by FPKM method [68]. The rigorous algorithm which described by Stephane Audic was used to identify differentially expressed genes between different samples [69], and False discovery rare(FDR) method was applied to analyze the threshold of the P-value in multiple test. We choose “FDR ≤ 0.001 and the absolute value of log2 Ratio ≥ 1” as the threshold to identify the significance of gene expression difference. All differentially expressed genes were performed to GO enrichment analysis, and were mapped to KEGG database to identify significantly enrich metabolic pathways or signal transduction pathways.

### Quantitative real-time PCR (qRT-PCR) analysis

To investigate the mRNA expression of putative genes involved in phenylethyl chromones biosynthesis and salt stress, qRT-PCR was performed using the Bio-Rad Real-time System and SYBR Green PCR Master kit according to the manufacturer’s instruction. Total RNA from different samples was reverse transcribed using the MLV kit (Sigma) according the manufacturer’s recommendations. 10 ng template cDNA was used in 20 μL PCR reaction mixture containing 10 μL fast SYBR Green master mix (Invitrogen) and 0.2 μM gene-specific primer (Additional file 7: Table S5; See supporting information). A DNA Engine Option 2 thermal cycler was carried out as the following program: one cycle of 95 °C for 3 min and 40 cycles of 95 °C for 30 s and 60 °C for 30 s. The expression of candidate genes was normalized to the *Actin* gene which was normally used as an internal control [4]. The relative gene expression level was normalized by rasing 2 to the power of the negative value of ∆∆Ct for each sample [70]. For all the qRT-PCR analysis, three biological experiments and three experimental replicates were calculated to analyze the candidate gene expression.

## Additional files

Additional file 1: Table S1. The length distribution of unigenes. (DOCX 18 kb) Additional file 2: Figure S1. Gene ontology (GO) functional enrichment analysis of assembled unigenes. A total of 53514 matched unigenes were classfied into three principal categories: biological process, cellular component and molecular function. (PPTX 165 kb) Additional file 3: Figure S2. COG functional classification of all unigenes sequence. 29 387(31.59 %) transcripts showed significant similarity to sequences in the COG databases and were clustered into 25 categories. (PPTX 213 kb) Additional file 4: Table S2. The KEGG analysis of all unigenes. (XLSX 17 kb) Additional file 5: Table S3. The KEGG analysis of differentially expressed genes at 24 h in salt-treated calli. (XLSX 20 kb) Additional file 6: Table S4. The KEGG analysis of differentially expressed genes at 120 h in salt-treated calli. (XLSX 19 kb) Additional file 7: Table S5. The specific primers of 26 candidate genes. (XLSX 15 kb) Additional file 8: Table S6. The number of differentially expression genes related to receptor kinase, MAPK pathway and Ca^2+^ signal pathway. (DOCX 22 kb) Additional file 9: Table S7. The number of differentially expressed genes related to transcription factors. (DOCX 20 kb) Additional file 10: Table S8. The number of differentially expressed ungenes involved in biosynthesis of 2-(2-phenylethyl)chromones. (DOCX 19 kb) Additional file 11: Figure S3 Phylogenetic tree analysis of plant type III PKSs. The tree was constructed by neighbor-joining algorithm and the reliability of the tree was measured by bootstrap analysis with 1000 replicates. The indicated scale represents 0.05 amino acid substitutions per site. (PPTX 150 kb)

## Abbreviations

BLAST: Basic Local Aligment Search Tool
CBF: CRT binding transcription factor
CHS: chalcone synthase
COG: clusters of orthologous groups of proteins
DEGs: differentially expression genes
FDR: false discovery rate
Go: gene ontology
KEGG: The KEGG resource for deciphering the genome
Nr: ncbi nr database
qPCR: quantitative real-time PCR
RNA-seq: RNA sequencing
TF: transcription factor
