# Supplementary material for: Salinity stress induces the production of 2-(2-phenylethyl)chromones and regulates novel classes of responsive genes involved in signal transduction in Aquilaria sinensis calli
Source: BMC Plant Biol. 2016 May 26;16:119. doi: 10.1186/s12870-016-0803-7 (PMC4881210; doi:10.1186/s12870-016-0803-7)
Supplement: Additional file 1: Table S1. — The length distribution of unigenes. (DOCX 18 kb) [file 12870_2016_803_MOESM1_ESM.docx]

**Table S1. The length distribution of unigenes**

| Samples | Number/percent | Length distribution of Unigenes | | | | |
| --- | --- | --- | --- | --- | --- | --- |
|  |  | 100-500nt | 500-1000 nt | 1000-1500nt | 1500-2000nt | ≥2000 nt |
| Control | Number | 36235 | 13013 | 10768 | 9841 | 21978 |
|  | Percent | 39.46% | 14.17% | 11.73% | 10.72% | 23.93% |
| Induced-24 h | Number | 34887 | 12184 | 9865 | 8965 | 17969 |
|  | Percent | 41.6% | 14.53% | 11.76% | 10.69% | 21.42% |
| Induced-120 h | Number | 35963 | 12423 | 10061 | 8698 | 16529 |
|  | Percent | 42.98% | 14.85% | 12.02% | 10.4% | 19.75% |
| All | Number | 24644 | 15349 | 12658 | 11953 | 28437 |
|  | Percent | 26.49% | 16.5% | 13.6% | 12.85% | 30.56% |
