# Supplementary figures and images for: Salinity stress induces the production of 2-(2-phenylethyl)chromones and regulates novel classes of responsive genes involved in signal transduction in Aquilaria sinensis calli
Source: BMC Plant Biol. 2016 May 26;16:119. doi: 10.1186/s12870-016-0803-7 (PMC4881210; doi:10.1186/s12870-016-0803-7)

## Slide 1
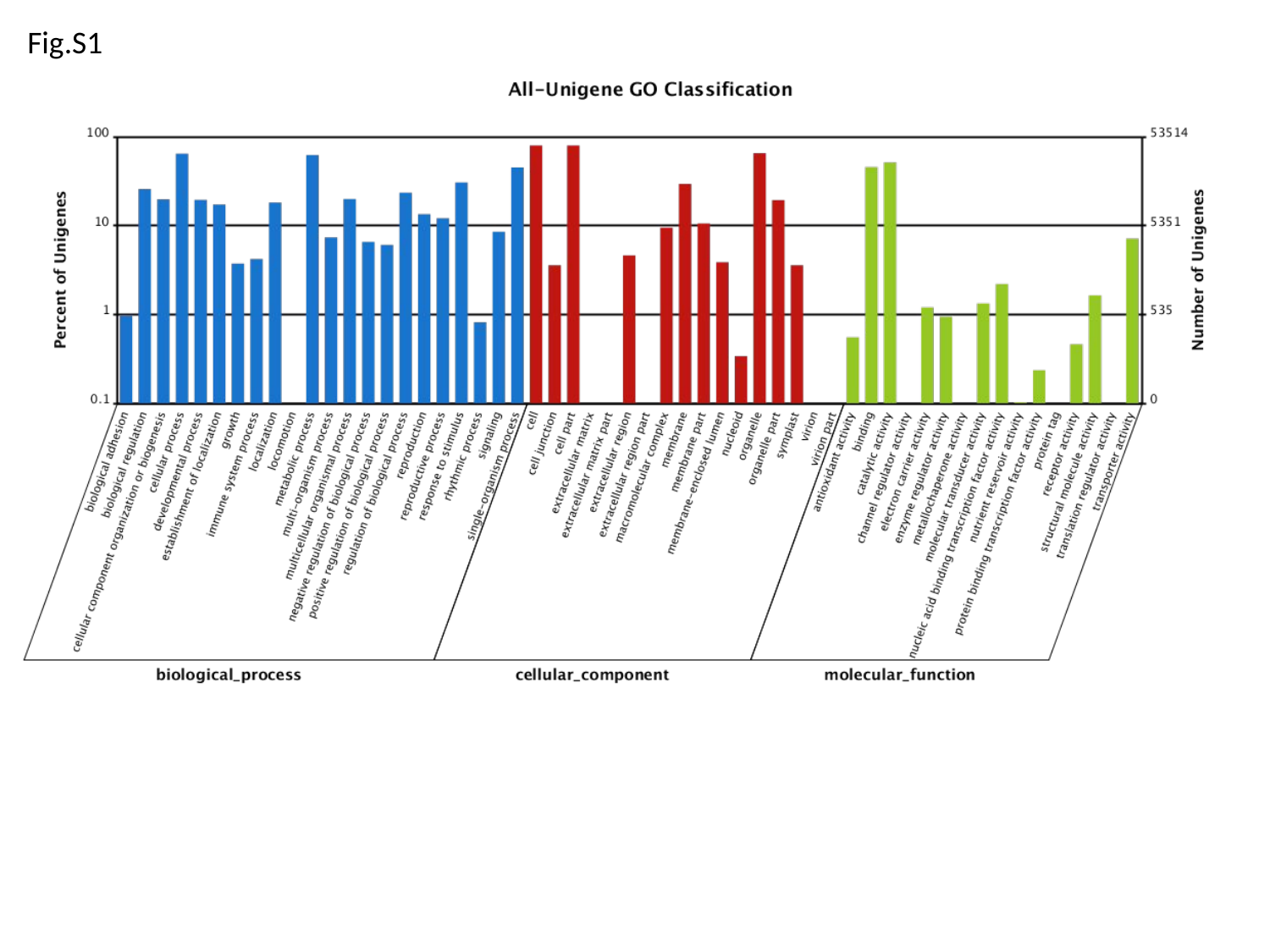

Fig.S1

Supplement: Additional file 2: Figure S1. — Gene ontology (GO) functional enrichment analysis of assembled unigenes. A total of 53514 matched unigenes were classfied into three principal categories: biological process, cellular component and molecular function. (PPTX 165 kb) [file 12870_2016_803_MOESM2_ESM.pptx]

## Slide 1
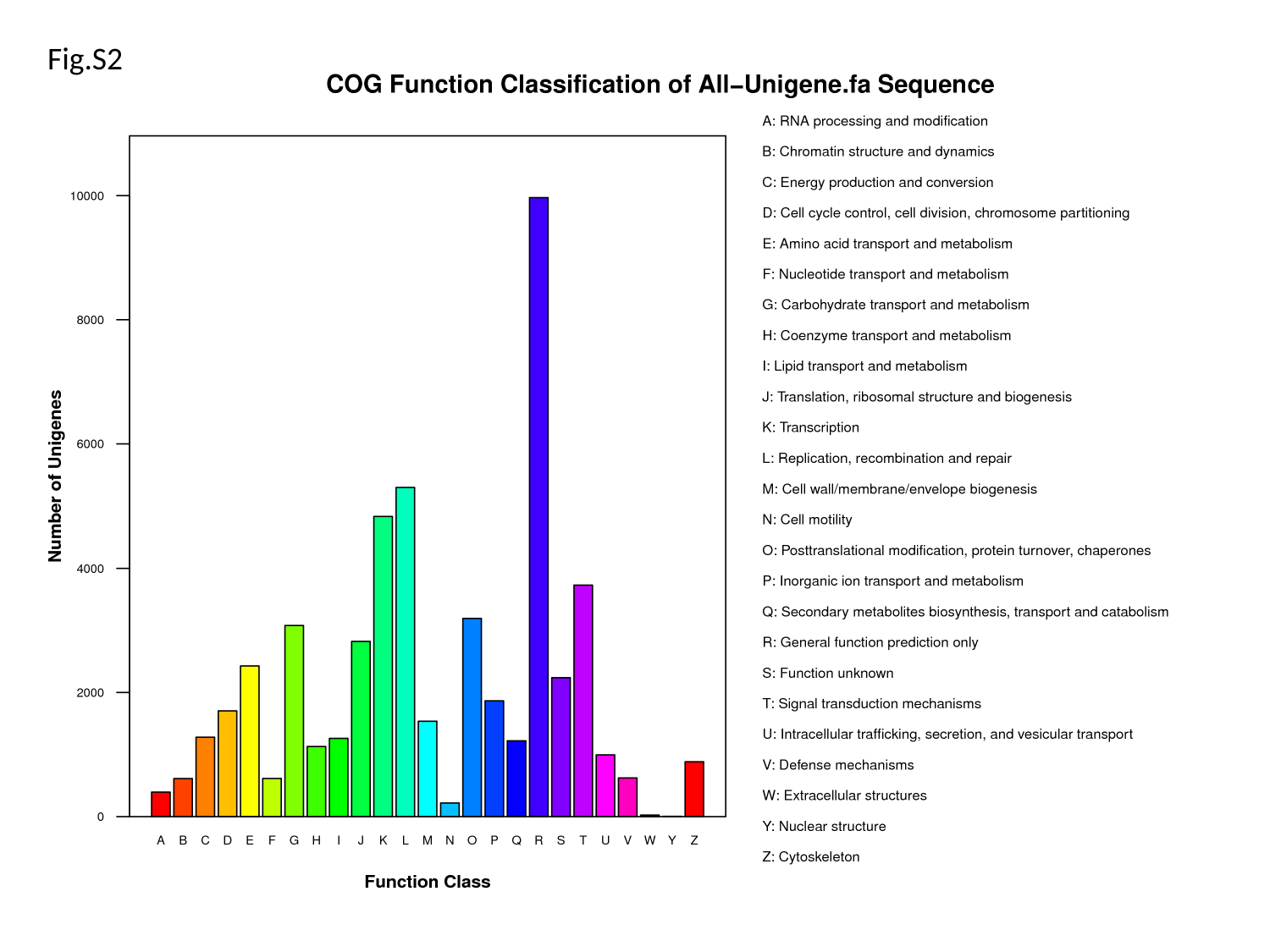

Fig.S2

Supplement: Additional file 3: Figure S2. — COG functional classification of all unigenes sequence. 29 387(31.59 %) transcripts showed significant similarity to sequences in the COG databases and were clustered into 25 categories. (PPTX 213 kb) [file 12870_2016_803_MOESM3_ESM.pptx]

## Slide 1
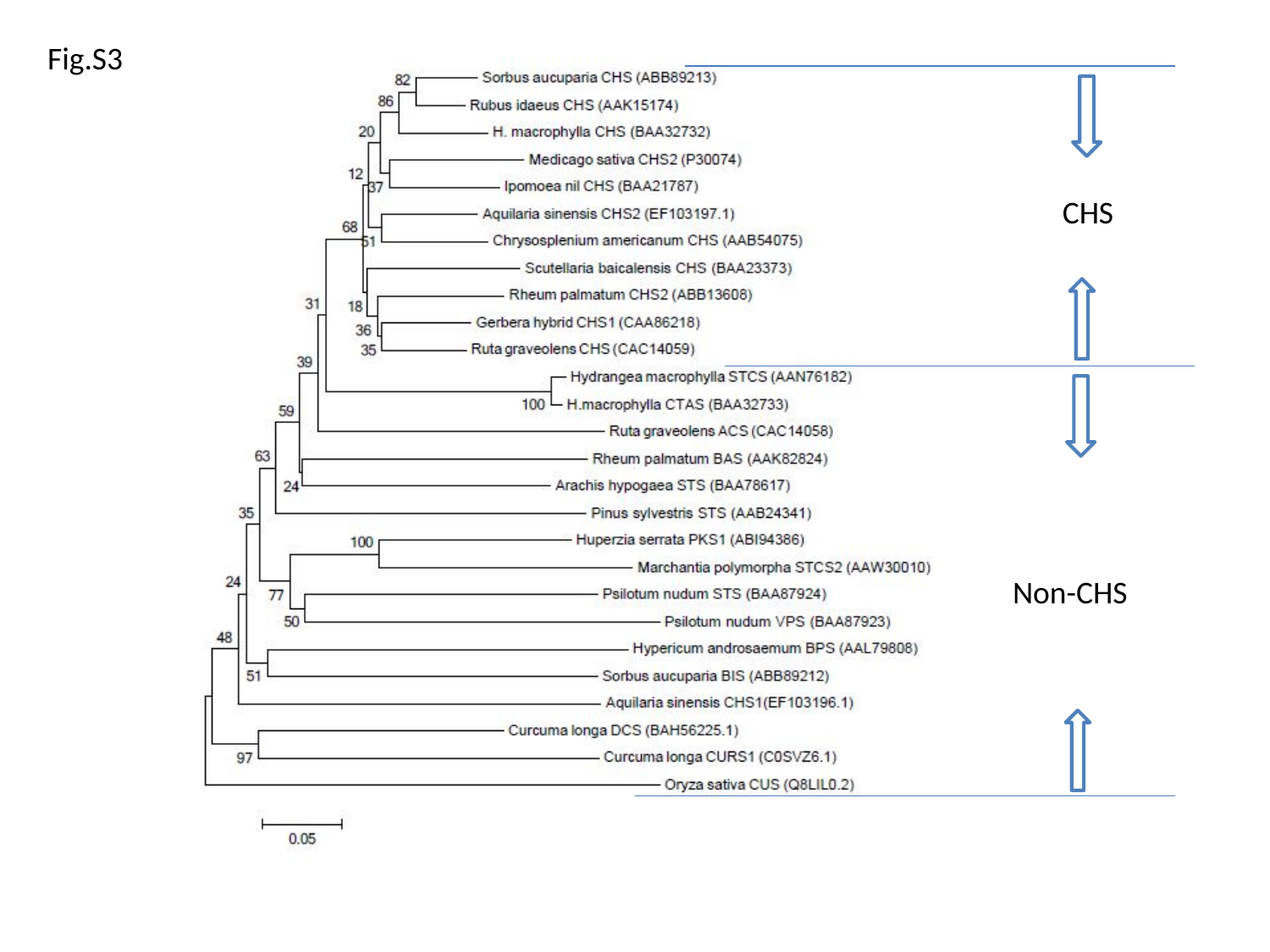

Fig.S3
CHS
Non-CHS

Supplement: Additional file 11: Figure S3 — Phylogenetic tree analysis of plant type III PKSs. The tree was constructed by neighbor-joining algorithm and the reliability of the tree was measured by bootstrap analysis with 1000 replicates. The indicated scale represents 0.05 amino acid substitutions per site. (PPTX 150 kb) [file 12870_2016_803_MOESM11_ESM.pptx]
