# Supplementary material for: Salinity stress induces the production of 2-(2-phenylethyl)chromones and regulates novel classes of responsive genes involved in signal transduction in Aquilaria sinensis calli
Source: BMC Plant Biol. 2016 May 26;16:119. doi: 10.1186/s12870-016-0803-7 (PMC4881210; doi:10.1186/s12870-016-0803-7)
Supplement: Additional file 8: Table S6. — The number of differentially expression genes related to receptor kinase, MAPK pathway and Ca2+ signal pathway. (DOCX 22 kb) [file 12870_2016_803_MOESM8_ESM.docx]

**Table S6. The number of differentially expression genes related to receptor-like kinases, MAPK pathway and Ca^2+^ signal pathway**

| family, group, or pathway | Number of genes | | | | |  |
| --- | --- | --- | --- | --- | --- | --- |
|  | **Total**  **DEGs** | **Induced-24h** | | **Induced-120h** | | **Co-**  **regulated** |
|  |  | **Up-**  **unigenes** | **Down-**  **unigenes** | **Up-**  **unigenes** | **Up-**  **unigenes** |  |
| Receptor-like kinase | **688** | **265** | **263** | **181** | **230** | **253** |
| LRR receptor-like serine/threonine-protein kinase(LRR-RLK) | 262 | 93 | 108 | 71 | 84 | 95 |
| Leucine-rich repeat receptor-like protein kinase FLS2 | 88 | 38 | 38 | 25 | 29 | 42 |
| Brassinosteroid insensitive 1-associated receptor kinase(BRI) | 16 | 5 | 5 | 7 | 5 | 6 |
| Proline-rich receptor-like protein kinase( PERK) | 92 | 31 | 39 | 22 | 36 | 36 |
| Cysteine-rich receptor-like protein kinase (CRR-RLK) | 42 | 31 | 7 | 19 | 5 | 20 |
| G-type lectin S-receptor-like serine/threonine-protein kinase (SRK) | 84 | 47 | 14 | 15 | 31 | 23 |
| Receptor-like protein kinase | 151 | 46 | 56 | 37 | 50 | 51 |
| Somatic embryogenesis receptor kinase | 13 | 1 | 12 | 1 | 11 | 12 |
| Wall-associated receptor kinase-like | 10 | 4 | 1 | 8 | 1 | 4 |
| L-type lectin-domain containing receptor kinase | 13 | 3 | 7 | 5 | 6 | 8 |
| Other receptor kinase | 21 | 9 | 19 | 3 | 6 | 4 |
| MAPK pathway | **26** | **12** | **11** | **3** | **8** | **9** |
| Mitogen-activated protein kinase kinase kinase | 16 | 10 | 5 | 3 | 2 | 4 |
| Mitogen-activated protein kinase kinase | 4 | 1 | 3 | 0 | 2 | 2 |
| Mitogen-activated protein kinase | 6 | 1 | 3 | 0 | 4 | 3 |
| Ca^2+^ signal pathway | **81** | **29** | **35** | **27** | **16** | **26** |
| Calmodulin | 23 | 9 | 7 | 7 | 6 | 6 |
| Calcium-binding protein (CML) | 29 | 7 | 14 | 16 | 6 | 14 |
| Calcium-dependent protein kinase(CDPK) | 20 | 12 | 7 | 4 | 3 | 6 |
| Calcineurin B-like protein | 9 | 1 | 7 | 0 | 1 | 0 |
