# Supplementary material for: Salinity stress induces the production of 2-(2-phenylethyl)chromones and regulates novel classes of responsive genes involved in signal transduction in Aquilaria sinensis calli
Source: BMC Plant Biol. 2016 May 26;16:119. doi: 10.1186/s12870-016-0803-7 (PMC4881210; doi:10.1186/s12870-016-0803-7)
Supplement: Additional file 9: Table S7. — The number of differentially expressed genes related to transcription factors. (DOCX 20 kb) [file 12870_2016_803_MOESM9_ESM.docx]

**Table S7. The number of differentially expressed genes related to transcription factors**

| Transcription factor family | Number of unigenes | | | | | | | |
| --- | --- | --- | --- | --- | --- | --- | --- | --- |
|  | **Total**  **DEGs** | **Induced-24 h** | | | **Induced-120 h** | | | **Co-**  **regulated**  **unigenes** |
|  |  | **Up-**  **unigenes** | | **Down-**  **unigenes** | | **Up-**  **unigenes** | **Down-**  **unigenes** |  |
| Ethylene-responsive transcription factor ERF( AP2/ERF ) | 73 | 48 | 17 | | 22 | | 16 | 30 |
| Transcription factor MYB | 71 | 49 | 13 | | 20 | | 14 | 24 |
| WRKY transcription factor | 58 | 43 | 3 | | 32 | | 7 | 27 |
| Transcription factor bHLH | 63 | 15 | 37 | | 7 | | 33 | 30 |
| Homeodomain-containing transcription factor ( homeobox) | 34 | 7 | 23 | | 4 | | 21 | 21 |
| NAC domain protein | 22 | 13 | 7 | | 5 | | 8 | 11 |
| Heat stress transcription factor | 17 | 15 | 1 | | 5 | | 0 | 4 |
| Transcription factor MYC | 26 | 14 | 9 | | 3 | | 8 | 9 |
| GRAS family transcription factor | 12 | 6 | 3 | | 4 | | 1 | 2 |
| Transcription factor TGA | 14 | 9 | 5 | | 3 | | 0 | 4 |
| Transcription factor TCP | 14 | 2 | 2 | | 0 | | 10 | 0 |
| GATA Transcription factor | 11 | 2 | 6 | | 1 | | 6 | 4 |
| Other transcription factors | 183 | 41 | 111 | | 22 | | 71 | 51 |
