# Supplementary material for: Salinity stress induces the production of 2-(2-phenylethyl)chromones and regulates novel classes of responsive genes involved in signal transduction in Aquilaria sinensis calli
Source: BMC Plant Biol. 2016 May 26;16:119. doi: 10.1186/s12870-016-0803-7 (PMC4881210; doi:10.1186/s12870-016-0803-7)
Supplement: Additional file 10: Table S8. — The number of differentially expressed ungenes involved in biosynthesis of 2-(2-phenylethyl)chromones. (DOCX 19 kb) [file 12870_2016_803_MOESM10_ESM.docx]

**Table S8. The number of differentially expressed ungenes involved in biosynthesis of 2-(2-phenylethyl)chromones**

| Family | Number of unigenes | | | | | |
| --- | --- | --- | --- | --- | --- | --- |
|  | **Total**  **DEGs** | **Induced-24 h** | | **Induced-120 h** | | **Co-**  **regulated**  **unigenes** |
|  |  | **Up-**  **unigenes** | **Down-**  **unigenes** | **Up-**  **unigenes** | **Down-**  **unigenes** |  |
| Chalcone synthase | 4 | 2 | 1 | 2 | 1 | 2 |
| Flavonol3-*O*-  methyltransferase | 21 | 8 | 8 | 10 | 7 | 12 |
| Caffeoyl-CoA *O*-methyltransferase | 8 | 5 | 2 | 4 | 1 | 4 |
